# Genetic Mutations and Non-Genomic Dysregulation in Human Preimplantation Embryo Arrest

**DOI:** 10.3390/ijms27052135

**Published:** 2026-02-25

**Authors:** Jianan Jiang, Junhua Peng, Lin Li, Min Xu

**Affiliations:** 1 Guangdong Provincial Key Laboratory of Proteomics, Department of Pathophysiology, School of Basic Medical Sciences, Southern Medical University, Guangzhou 510515, China; 2 Obstetrics and Gynecology Center, Zhujiang Hospital, Southern Medical University, Guangzhou 510282, China; 3 Department of Cell Biology, School of Basic Medical Sciences, Southern Medical University, Guangzhou 510515, China

**Keywords:** preimplantation embryo arrest (PREMBA), maternal-to-zygotic transition (MZT), zygotic genome activation (ZGA), epigenetic reprogramming

## Abstract

Human preimplantation embryo arrest (PREMBA) represents a significant clinical hurdle in assisted reproductive technology (ART), in which approximately 10% of in vitro fertilized (IVF) embryos arrest at the cleavage stages. Whole-exome sequencing (WES) studies have discovered numerous genetic mutations associated with preimplantation embryo arrest. These mutations often disrupt critical biological milestones such as maternal mRNA clearance (*BTG4*, *ZFP36L2*, *ZAR1*), subcortical maternal complex (*TLE6*, *PADI6*, *OOEP*, *NLRP2*, *NLRP5*, *NLRP7*, *KHDC3L*), DNA double-strand break formation and homologous recombination (*REC114*, *TOP6BL*, *MEI1*, *MEI4*, *TRIP13*), spindle assembly (*TUBB8* and *TUBA4A*) and cell cycle and checkpoints (*FBXO43*, *MOS*, *CHEK1*, *TRIP13*, *CDC20*), as well as nuclear transport and translational regulation (*KPNA7*, *DDOST*). However, the cause of most clinical cases remains genetically unexplained. Studies investigating these unexplained arrests have uncovered widespread multi-omics abnormalities, including transcriptional arrest, DNA hypermethylation, higher chromatin accessibility, aberrant histone modification, chromosomal aneuploidy and senescent-like states. This review provides a comprehensive overview of the molecular mechanisms underlying PREMBA, categorized into those that are attributable to known genetic mutations and those with unexplained reasons.

## 1. Introduction

The successful progression of a human embryo from a zygote to a blastocyst is a prerequisite for human reproduction, yet it is a remarkably inefficient process. In clinical IVF, a large proportion of preimplantation embryos experience developmental arrest at the cleavage stages. This phenomenon, termed as PREMBA, accounts for approximately 10% of cleavage-stage failures and poses a major economic and psychological burden on patients facing recurrent ART failure [1].

**Figure 1:**
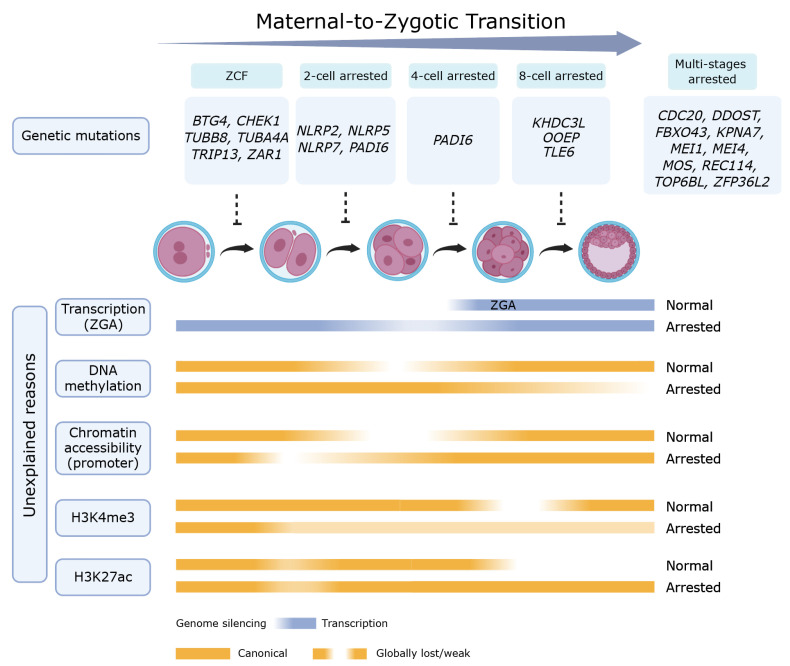
Schematic diagram illustrating the genetic mutations and multi-omics dysregulation associated with human PREMBA. Embryonic development undergoes a maternal-to-zygotic transition between the zygote and early blastocyst stages, encompassing maternal transcript clearance and zygotic genome activation. Genetic mutations disrupting this process can lead to stage-specific or multi-stage PREMBA (top), while non-genomic mechanisms involve dysregulation across multiple omics layers, including transcriptionally arrested, aberrant DNA hypermethylation, altered chromatin accessibility, and abnormal histone modifications (bottom). Active genome regions (transcribed or with canonical epigenomic marks) are shown in solid colors, while silenced regions (or globally lost/weak epigenomes) are represented by a color-to-white gradient. The full names of the genes have been described in the manuscript. ZCF, zygotic cleavage failure (created with Biorender.com).

The genetic basis of PREMBA is increasingly recognized as a Mendelian disorder. Current studies have identified 24 known causative genes associated with preimplantation embryo arrest [2,3] (Table 1 and Figure 1). These genes are critical for embryogenesis: notably, the maternal-to-zygotic transition (MZT), when developmental control shifts from the maternal factors to the embryonic genome. Dysregulators of this handover were categorized into functional clusters such as zygotic cleavage failure (ZCF), subcortical maternal complex (SCMC) deficiency and impaired nuclear transport—directly leading to embryonic arrest. For instance, mutations in Karyopherin subunit α7 (*KPNA7*) impair the nuclear import of proteins required for zygotic genome activation (ZGA) [4], while dominant mutations in checkpoint kinase 1 (*CHEK1*) explain the arrest at the first mitotic division [5].

Morphologically normal embryos often arrest due to MZT failure, characterized by impaired maternal transcript clearance and disrupted ZGA. This transcriptomic failure in arrested embryos is associated with the deficient expression level of primate-specific genes such as divergent paired-related homeobox (*DPRX*), arginine-fifty homeobox (*ARGFX*) [6] and the key transcription factor Yin Yang 1 (YY1) [7]. These blocks are coupled with epigenetic reprogramming defects, including metabolic genes (e.g., solute carrier family 2 member 3, (*SLC2A3*)) silenced by DNA hypermethylation status [8], aberrant histone 3 lysine 27 acetylation (H3K27ac) retention and transposable element de-repression [6]. Ultimately, these failures force embryos into a senescent-like state marked by metabolic collapse and Aurora A kinase (AURKA) repression, which induce microtubule cytoskeletal distortion and impaired glycolysis [7].

This review aims to synthesize current evidence of the genetic reasons and the multi-dimensional omics perturbation underlying human PREMBA, providing a framework for precision diagnosis and potential rescue strategies in the future.

## 2. Genetic Mutations Leading to Preimplantation Embryo Arrest

Here, we provide a comprehensive review of the genetic mutations and pathogenic mechanisms of human preimplantation embryo arrest, focusing on developmental failure from the zygote stage to the blastocyst stage. Based on previous studies, the genetic etiology is categorized into six functional groups (Table 1).

### 2.1. Maternal mRNA Decay and MZT

Zygotic cleavage failure represents those embryos that arrest at the one-cell stage, primarily due to defects in the MZT. B-cell translocation gene-4 (*BTG4*) encodes a critical maternal factor that recruits the CCR4–NOT deadenylase complex by bridging its catalytic subunit CCR4-NOT transcription complex subunit 7 (CNOT7) to initiate programmed maternal mRNA decay [9]. In 2020, Zheng et al. found the *BTG4* variant c.166G>A (p.Ala56Thr) disrupted BTG4-CNOT7 binding. Subsequent single-cell RNA-seq analysis of zygotes from patients with *BTG4* mutations and *Btg4* knockout mice revealed that 471 conserved maternal genes fail to degrade [10]. Mutations in *BTG4* prevent the clearance of maternal transcripts, leading to transcript accumulation, thereby blocking the transition to the zygotic program and causing zygotic arrest. Furthermore, ZFP36 ring finger protein like 2 (ZFP36L2), an RNA-binding protein that acts as an adapter for CCR4-NOT transcription complex subunit 6 like (CNOT6L) to facilitate maternal mRNA degradation. Deficiency in this protein leads to the abnormal retention of transcripts and recurrent preimplantation arrest [11].

Zygote Arrest 1 (*ZAR1*) as a mRNA-binding protein (RBP) mediates maternal mRNA storage by forming the mitochondria-associated ribonucleoprotein domain (MARDO), a liquid–liquid phase-separated compartment that translationally represses mRNAs [12,13,14]. It is one of the maternal-effect genes that is critical for mediating the MZT and most embryos with *Zar1* knockout arrest at the one-cell stage in mice [15]. In 2020, Tian et al. identified two variations (one novel synonymous variation (c.516C>T) and one novel intron variation (c.964-55A>T)) in arrest zygotes. However, the subsequent analysis found both mutations in *ZAR1*, with no effect on ZAR1 protein function [16]. A recent study identified three functional variations in *ZAR1* from two patients. The *ZAR1* variant c.353T>C (p.V118A) disrupts MARDO localization and compromises both oocyte maturation and maternal mRNA storage, while *ZAR1* mutations c.1190G>A (p.R397Q) and c.362C>A (p.S121*) abolish RNA-binding capacity [17]. This finding elucidated the mechanism of *ZAR1* mutations leading to human zygotic arrest.

### 2.2. Subcortical Maternal Complex (SCMC)

The SCMC is a multi-protein complex, which is unique to mammalian oocytes and early embryos, that regulates symmetric cleavage, F-actin dynamics, and ZGA [18]. The core structure of the human SCMC comprises eight essential components: peptidyl arginine deiminase 6 (PADI6), transducin-like enhancer of split 6 (TLE6), oocyte expressed protein (OOEP), KH domain containing 3 like (KHDC3L), NLR family pyrin domain containing 2 (NLRP2), NLR family pyrin domain containing 5 (NLRP5), NLR family pyrin domain containing 7 (NLRP7) and zinc finger BED-type containing 3 (ZBED3) [19]. Notably, pathogenic variants in *PADI6* [20], *TLE6* [21], *OOEP* [22], *KHDC3L* [23], *NLRP2/5* [24], and *NLRP7* [23,25] have been demonstrated to cause early embryonic arrest.

PADI6 is predominantly localized in cytoplasmic lattices (CPLs)—highly specialized cytoskeletal structures that store ribosomes and maternal proteins in mature oocytes [26]. In 2016, Xu et al. identified a homozygous *PADI6* nonsense mutation (c.1141C>T [p.Gln381*]) and two affected individuals with compound heterozygous mutations (c.2009_2010del [p.Glu670Glyfs*48] and c.633T>A [p.His211Gln]; c.1618G>A [p.Gly540Arg] and c.970C>T [p.Gln324*]) in a family [20]. Embryos from these patients showed significantly reduced levels of phosphorylated RNA polymerase II and impaired transcription of ZGA-related genes, which typically arrest between the 2- and 5-cell stages [20].

Another essential component, TLE6, facilitates SCMC assembly and organizes the F-actin cytoskeleton. Missense mutations in *TLE6* affect protein phosphorylation and cause a termination at meiosis II, while homozygous frameshift mutations were with similar phenotypes in the *Tle6* knockout mouse, whose embryos undergo initial cleavage with severe fragmentation and arrest before morula formation [21]. Biallelic mutations in *NLRP2*, *NLRP5*, and *NLRP7* further destabilize the SCMC, often resulting in 2-cell stage arrest or the development of hydatidiform moles due to defects in the establishment of maternal imprinting marks [23,24]. Interestingly, clinical data show significant phenotypic heterogeneity; some patients with modest functional disruption of *NLRP2/5* can produce a limited number of viable embryos and even give birth to healthy babies after multiple attempts, highlighting the variable penetrance of these variants.

Furthermore, *KHDC3L* (*FILIA*) and *OOEP* protect the genomic stability of the early embryo. *KHDC3L* encodes a protein that interacts with NLRP7 and plays a crucial role in maintaining euploidy during cleavage-stage embryogenesis by modulating spindle assembly [23]. Although lack of OOEP modestly affected zygotic genome activation in human early embryos. Their mutations lead to severe aneuploidy and apoptosis, typically terminating embryogenesis at the morula stage [22,23].

### 2.3. DNA Double-Strand Break and Homologous Recombination

Errors in genes required for DNA double-strand break (DSBs) and homologous recombination were often manifested as post-fertilization developmental failure. Genes such as REC114 meiotic recombination protein (*REC114*) and meiotic double-stranded break formation protein 1/4 (*MEI1/4*) are essential for the formation of DSBs and proper chromosome synapsis during meiosis [27,28]. REC114 dimerizes and partners with MEI4 in a 2:1 heterotrimer, forming a regulatory complex with IHO1 to orchestrate meiotic DSBs [29,30]. Pathogenic variants in these genes lead to abnormal spindle formation and severe aneuploidy, resulting in multiple pronuclei or early embryo arrest during the cleavage stages. In two independent families, *REC114* homozygous mutations were associated not only with embryonic arrest but also with the implantation failure of the few surviving embryos, suggesting that even embryos that bypass the initial cleavage block are genetically too compromised to establish a pregnancy [31].

Thyroid hormone receptor interaction protein 13 (TRIP13) critically regulates meiotic chromosome recombination by mediating meiotic checkpoint complex dissociation [32]. Huiling et al. identified three *TRIP13* missense variants—c.1141G>A (p.Glu381Lys), c.77A>G (p.His26Arg), and c.1258A>G (p.Lys420Glu)—in two patients, which were found to contribute to zygotic checkpoint failure. All three variants disrupted hydrogen bonding patterns and consistently elevated DNA damage levels. Subsequent RNA-seq analysis revealed significant upregulation of DNA repair-related genes in both germinal vesicle (GV) oocytes and zygote embryos [33].

Additionally, mutations in TOP6B like initiator of meiotic double strand breaks (*TOP6BL*) and members of its complex have been associated with both embryonic arrest and the development of hydatidiform moles, which cause the failure of programmed DSB formation and subsequently meiotic defects, highlighting the importance of genomic integrity from the very first zygotic division [3].

### 2.4. Spindle Assembly

Microtubule integrity is largely maintained by tubulin isotypes; large-scale analysis of de novo mutations (DNMs) identified Tubulin Alpha 4a (*TUBA4A*) as a high-risk gene. TUBA4A is a specialized α-tubulin isoform distinguished by its unique C-terminal structure, atypical post-translational modification properties, and tissue-enriched expression (notably in the central nervous system, platelets, and muscle) [34]. Mutations in *TUBA4A* exert dominant negative effects that destabilize the microtubule network and prevent proper spindle formation in oocytes or early embryos [35].

Mutations in Tubulin Beta 8 class VIII (*TUBB8*) primarily cause oogenesis arrest at the MI stage, while some variants allow fertilization but cause the embryos to subsequently arrest during the first few cell divisions [11,36]. *TUBB8* mutations disrupt microtubule dynamics and meiotic spindle assembly in oocytes, leading to variable fertilization outcomes and embryonic arrest, even though some homozygous mutations paradoxically permit spindle formation despite the complete loss of functional protein [37].

Furthermore, *TUBB8* deficiency may impair maternal mRNA clearance. Compared to random primer-based transcription, the reverse transcription efficiency of oligo-dT-mediated reactions was significantly reduced in oocytes with *TUBB8* variants, suggesting that longer poly(A) tails persist in the affected embryos [9].

### 2.5. Cell Cycle and Checkpoints

Rapid embryonic divisions require robust cell cycle control and stable microtubule structures. F-Box protein 43 (FBXO43, also called EMI2) acts as an inhibitor of the anaphase-promoting complex/cyclosome (APC/C) to stabilize Cyclin B1. Its loss-of-function variants fail to stabilize Cyclin B1, leading to a premature exit from the meiotic or early mitotic stages and causing preimplantation embryo arrest characterized by severe fragmentation [38]. In mouse experiments, supplementation with wild-type human FBXO43 cRNA can prevent the spontaneous parthenogenetic activation caused by *Fbxo43* knockdown, whereas patient-derived mutant cRNAs show a significantly reduced ability to restore cell cycle arrest [38].

MOS proto-oncogene (MOS), a kinase in the extracellular-signal-regulated kinase (ERK) pathway, is required for mitochondrial function and F-actin assembly; biallelic mutations in *MOS* lead to severe embryo fragmentation and the production of unusually large polar bodies [39]. New genomic evidence also points to cyclin N-terminal domain containing 2 (*CNTD2*) and Speedy/RINGO cell cycle regulator family member C (*SPDYC*), which are involved in cyclin-dependent kinase phase transitions during the mitotic cell cycle; overexpression of mutant *Cntd2* in mouse zygotes leads to embryonic arrest [3].

Cell division cycle 20 (CDC20) acts as a coactivator of the APC/C during mitosis, regulating spindle assembly checkpoint (SAC) activity to ensure genome stability [40]. *CDC20* mutations were found to cause oocyte maturation arrest, fertilization failure, and early embryonic arrest in five infertile individuals [40,41,42].

CHEK1 (also known as CHK1) is a cell cycle checkpoint kinase in which gain-of-function mutations enhance its kinase activity, resulting in an abnormal accumulation of inhibitory phosphorylated cyclin dependent kinase 1 (CDK1). This cascade blocks the G2/M transition and arrests the zygotes at the pronucleus stage through pronuclear fusion failure. Research has shown that this arrest can be specifically rescued by the CHEK1 inhibitor PF477736, which restores the kinase activity to a normal level and allows the zygote to recover through a mitosis process and develop into a high-quality blastocysts [5]. Additionally, cell cycle progression was significantly downregulated in both GV oocytes and zygote embryos from *TRIP13* variant patients [33].

### 2.6. Nuclear Transport and Translational Control

Precise embryonic development requires the nuclear import of maternal factors to initiate the ZGA program. Karyopherin subunit α7 (KPNA7) is the most abundant human karyopherin in oocytes; its variants reduce protein levels and specifically impair its capacity to bind its substrate, ribosomal L1 domain containing 1 (RSL1D1) [4]. This prevents the nuclear transport of factors that are essential for starting zygotic transcription, leading to ZGA failure and PREMBA. Comparative studies between humans and mice revealed an evolutionary functional shift: in mice, *Kpna2* (rather than *Kpna7*) is the primary carrier for RSL1D1, and *Kpna2* knockout mice accurately recapitulate the human *KPNA7* deficiency phenotype, including a 2-cell stage arrest and transcription termination [4]. Microinjection of high concentrations of human KPNA7 complementary RNA (cRNA) into *Kpna2* deficient mouse zygotes can successfully restore RSL1D1’s nuclear entry and rescue its development to the blastocyst stage [4].

Additionally, recent genomic burden tests have identified the novel candidate gene dolichyl-diphosphooligosaccharide-protein glycosyltransferase non-catalytic subunit (*DDOST*), encoding a subunit of the oligosaccharyltransferase complex involved in protein N-glycosylation [3]. Functional studies in HeLa cells and mouse oocytes showed that variants in *DDOST* alter the subcellular localization of the protein and cause a significant reduction in the polar body extrusion rate, linking glycosylation defects to abnormal fertilization and early embryo developmental arrest [3].

Collectively, these established causative genes delineate the critical pathways essential for embryonic development. As summarized in Table 1, mutations in these genes will lead to early embryonic arrest. These genes are involved in multiple biological processes, including maternal mRNA decay and MZT failure (*BTG4*, *ZAR1*, *ZFP36L2*), subcortical maternal complex formation (*TLE6*, *PADI6*, *OOEP*, *NLRP2/5/7*, *KHDC3L*), DNA double-strand break and homologous recombination (*REC114*, *TOP6BL*, *MEI1/4*, *TRIP13*), spindle assembly (*TUBB8*, *TUBA4A*), cell cycle regulation and checkpoint control (*MOS*, *FBXO43*, *TRIP13*, *CHEK1*, *CDC20*), and nuclear transport and translational regulation (*KPNA7*, *DDOST*).

**Table 1:** Genetic mutations that lead to early embryonic arrest.

Type	Arrested Stage	Related Genes
Maternal mRNA decay and MZT	Zygote	*BTG4*, *ZAR1*
	Multi-stages	*ZFP36L2*
Subcortical maternal complex (SCMC)	2-cell	*NLRP2*, *NLRP5*, *NLRP7*, *PADI6*
	4-cell	*PADI6*
	8-cell	*TLE6*, *OOEP*, *KHDC3L*
DNA double-strand break and homologous recombination	Zygote	*TRIP13*
	Multi-stages	*REC114*, *MEI1*, *MEI4*, *TOP6BL*
Spindle assembly	Zygote	*TUBB8*, *TUBA4A*
Cell cycle and checkpoints	Zygote	*CHEK1*, *TRIP13*
	Multi-stages	*FBXO43*, *MOS, CDC20*
Nuclear transport and translational control	Multi-stages	*KPNA7*, *DDOST*

*BTG4*, B-cell translocation gene-4; *ZAR1*, zygote arrest 1; *ZFP36L2*, ZFP36 ring finger protein like 2; *NLRP2*, NLR family pyrin domain containing 2; *NLRP5*, NLR family pyrin domain containing 5; *NLRP7*, NLR family pyrin domain containing 7; *PADI6*, peptidyl arginine deiminase 6; *TLE6*, transducin-like enhancer of split 6, subcortical maternal complex member; *OOEP*, oocyte expressed protein; *KHDC3L*, KH domain containing 3 like, subcortical maternal complex member; *TRIP13*, thyroid hormone receptor interactor 13; *REC114*, REC114 meiotic recombination protein; *MEI1*, meiotic double-stranded break formation protein 1; *MEI4*, meiotic double-stranded break formation protein 4; *TOP6BL*, TOP6B like initiator of meiotic double strand breaks; *TUBB8*, tubulin beta 8 class VIII; *TUBA4A*, tubulin alpha 4a; *CHEK1*, checkpoint kinase 1; *TRIP13*, thyroid hormone receptor interactor 13; *FBXO43*, F-Box protein 43; *MOS*, MOS proto-oncogene, serine/threonine kinase; *CDC20*, cell division cycle 20; *KPNA7*, karyopherin subunit alpha 7; and *DDOST*, dolichyl-diphosphooligosaccharide-protein glycosyltransferase non-catalytic subunit.

## 3. Human Embryonic Arrest Due to Unexplained Reasons

Although numerous mutations linked to preimplantation embryonic arrest have been identified, a substantial proportion of cases were classified as embryonic arrest with unexplained reasons, which means arrest with non-genetic causes. Current studies on these embryos primarily focuses on biological processes related to MZT and ZGA. The following section provide an in-depth summary of molecular mechanisms underlying unexplained human preimplantation embryo arrest, categorized by omics dimensions.

### 3.1. Transcriptomic Dysregulation of MZT

In the development from growing oocytes (GO) to the GV stage, maternal mRNAs form the fundamental basis for oocyte maturation. Following meiotic resumption, maternal transcripts undergo massive degradation—termed as maternal RNA decay (M-decay), which continues through the zygotic stage to ensure proper MZT [43].

Failure of M-decay often leads to zygotic arrest. Notably, Sha et al. observed impaired M-decay in human arrested zygotes (five out of seven cases) in mutation-unidentified patient (unid-patient) [44]. Subsequent single-cell RNA-seq analysis found that nearly 50% (1490/3179) of maternal mRNAs, which were degraded in normal embryos during the GV-to-zygote transition were stabilized in unid-patient embryos [44]. Reverse transcription quantitative polymerase chain reaction (RT-qPCR) results indicate that the expression level of *CNOT6L*, *CNOT7*, and *BTG4* were significantly lower in human embryos with M-decay defects [44]. These findings demonstrate the impaired BTG4-CCR4-NOT deadenylation pathway in arrested embryos.

Successful development requires the sequential removal of maternal transcripts through M-decay and zygotic (Z)-decay pathways [44]. Factors such as BTG4 and CNOT6L are essential for M-decay, while the YAP1-TEAD4 complex triggers Z-decay by activating factors like terminal uridylyl transferase 4/7 (TUT4/7). In arrested embryos, the expression level of these factors is often significantly decreased, leading to the abnormal accumulation of maternal transcripts [44].

Furthermore, recent studies revealed that microtubule-associated protein 1 light chain 3 Beta (LC3B)-mediated mRNA decay exhibits significantly faster degradation kinetics compared to the canonical BTG4-CCR4-NOT pathway [45]. LC3B is a maternal RNA-binding protein which mediates mRNA degradation prior to autolysosome formation [46]. Liu et al. found that knockdown of *LC3B* or inhibition of autophagy significantly delayed maternal mRNA clearance, consequently disrupting ZGA. Subsequent mechanistic analysis identified the maternal suppressor of variegation 3–9 homolog 2 (*Suv39h2*) as a key LC3B-targeted gene, with its aberrant persistence demonstrating a significant correlation with embryonic developmental failure [45].

Human ZGA occurs in two waves: minor ZGA at the zygote at the 4-cell stage and major ZGA around the 8-cell stage [47]. The failure to activate critical PAIRED (PRD)-like homeobox transcription factors, particularly DPRX and ARGFX, disrupts ZGA and leads to 8-cell arrest. These factors are primate-specific and are expressed exclusively during preimplantation development [6]. Their deficiency leads to catastrophic failure in major ZGA and impairs the transcription of subsequent lineage specification markers such as nanog homeobox (*NANOG*), GATA binding protein 3 (*GATA3*), and GATA binding protein 6 (*GATA6*). Furthermore, the zinc-finger protein YY1 acts as a master regulator of human ZGA; its knockdown results in mitosis-independent developmental delay and the silencing of thousands of zygotic genes, effectively halting the embryo’s progression to the morula stage [7].

### 3.2. Abnormal DNA Methylation Reprogramming

DNA methylation reprogramming is essential for human preimplantation embryo development. Global DNA demethylation occurred from the late zygote to the 2-cell stage, which depends on ten-eleven translocation (TET) enzymes and is an important prerequisite for successful ZGA [48]. Arrested embryos frequently display DNA hypermethylation states, retaining significantly higher levels of 5-methylcytosine (5mC) and 5-hydroxymethylcytosine (5hmC) [7,49].

The failure of TET-mediated active demethylation or the aberrant maintenance of methylation at promoter regions leads to hypermethylation states in the promoter regions of genes [49]. The embryos arrested at the 2-cell or 4-cell stage exhibit a higher DNA methylation level and a concomitantly elevated expression level of genes encoding key methylation enzymes, including DNA methyltransferase 1 (*DNMT1*) and ubiquitin like with PHD and ring finger domains 1 (*UHRF1*) [50]. Furthermore, using non-invasive time-lapse imaging, it has been demonstrated that normal 4-cell embryos possess lower 5mC levels than their abnormally developed or arrested counterparts, indicating that successful epigenetic reprogramming is a prerequisite for embryogenesis [49].

DNA hypermethylation is often found in intragenic regions that may contribute to mitosis-independent ZGA in arrested embryos. For instance, the glucose transporter SLC2A3 is frequently hypermethylated and downregulated in arrested embryos, directly linking epigenetic failure to metabolic collapse [7]. Interestingly, although normal embryogenesis shows a second wave of demethylation in the inner cell mass (ICM) at the blastocyst stage, arrested embryos remain locked in a rigid, highly methylated state [49].

### 3.3. Higher Chromatin Accessibility and Transposable Elements De-Repression

In arrested embryos, the reprogramming of chromatin accessibility was earlier than that of the transcriptome and DNA methylome. Those embryos show an independent gain of chromatin accessibility without cell division, leading to the formation of abnormal nucleosome-depleted regions (NDRs). The correlation between chromatin accessibility and transcription is higher in arrested embryos than that in normal zygote to 4-cell embryos. This premature chromatin accessibility is particularly prevalent in the promoters of major ZGA genes. The upregulated major ZGA genes in arrested zygote to 4-cell embryos were linked with the increased chromatin accessibility of their promoters. YY1 was highly enriched in arrested-specific proximal NDRs, which regulates major ZGA, not only in arrested zygotes to 4-cell embryos but also in normal 8-cell embryos [7].

Retro-transposable elements (retro-TEs) exhibit significant transcriptional activity during early embryogenesis and represent a major component of stage-specific gene expression during ZGA, such as interspersed nuclear elements (LINEs), short interspersed nuclear elements (SINEs), and endogenous retroviruses (ERVs) [51,52]. A recent study identified that the reduced expression level of MLT2A1, a human totipotency-specific long terminal repeat (LTR) of the ERVL subfamily, is linked to the developmental arrest of human embryos at the 8-cell stage. MLT2A1 mainly generates noncoding chimeric RNAs by fusion with diverse downstream sequences. The MLT2A1 conserved 5′ domain recruits heterogeneous nuclear ribonucleoprotein U (HNRNPU) to enhance RNA polymerase II activity, while heterogeneous 3′ fusion sequences expand its genomic targeting capacity, thereby orchestrating global ZGA during human embryogenesis [53].

Additionally, the aberrant openness results in the massive de-repression of transposable elements (TEs), such as the Alu, SINE/variable number of tandem repeats/Alu (SVAs), and LINE-1 families [7]. The activation of these elements is a hallmark of genomic instability and is strongly associated with increased DNA damage, as evidenced by a high frequency of γ-H2AX foci in arrested nuclei [7]. Furthermore, topologically associating domains (TADs) are established during human ZGA [54]. However, the downregulation of the CCCTC-binding factor (CTCF) may prevent the establishment of a functional higher-order chromatin architecture in arrested 8-cell embryos [7].

### 3.4. Defective Dynamics of Histone Modification

The precise remodeling of histone marks, particularly the erasure of parental marks and the establishment of zygotic ones, is essential for ZGA [50]. Human oocytes and early embryos utilize a broad, non-canonical histone H3 lysine 4 trimethylation (H3K4me3) pattern to maintain genomic silence; successful ZGA requires the transition of this mark to a precise, canonical pattern [55,56]. Arrested embryos often fail this transition due to the overexpression of the lysine demethylase 5B (*KDM5B*) or the loss of YY1-mediated recruitment, which prevents the proper activation of zygotic genes [7].

Histone H3 lysine 9 trimethylation **(**H3K9me3) is progressively established during human preimplantation development, exhibiting stage-specific patterns [57,58]. Aberrant H3K9me3 remodeling leads to the failure of major ZGA. Lysine demethylase 4a (*Kdm4a*) knockout mouse embryos exhibit a severe developmental delay or arrest at the 2-cell stage [59]. Notably, *Suv39h2* encodes a methyltransferase that is responsible for H3K9me3 modification, which was highly expressed in oocytes, followed by progressive downregulation during early embryogenesis. Liu et al. demonstrated that ectopic overexpression of *Suv39h2* in zygotes leads to developmental arrest and significantly impairs blastocyst formation [45].

The dynamics of H3K27ac are critical for preimplantation embryo development, which was broadly distributed across promoters. These marks are either removed or resolve into narrow peaks after ZGA [60]. Crucially, *DPRX* and *ARGFX* deficiency lead to the aberrant retention of H3K27ac [6]. Under normal conditions, deacetylases such as histone deacetylase 1 (HDAC1), histone deacetylase 2 (HDAC2), and sirtuin 1 (SIRT1) are activated to erase H3K27ac marks from 4-cell-specific genes to allow the transition into the 8-cell transcriptomic program. In arrested or *DPRX* and *ARGFX* double knockdown (dKD) embryos, *HDAC1* is significantly downregulated, causing prolonged activation of genes such as Leucine Twenty Homeobox (*LEUTX*), cyclin A1 (*CCNA1*), lysine demethylase 4E (*KDM4E*), and PRAME family member 2 (*PRAMEF2*) that were highly expressed in normal 4-cell embryos. This retention traps the embryo into an outdated developmental state. Interventions using the HDAC1 activator exifone have been shown to reduce H3K27ac levels and partially rescue the compaction and developmental potential of these embryos [6].

### 3.5. Metabolic Shifts, Cytoskeletal Distortion and Senescence

Physiological arrest is ultimately grounded in a senescent-like state and the collapse of the microtubule cytoskeleton. Arrested embryos exhibit a downregulation of ribosome- and histone-related genes, alongside the activation of the p53/p21 pathway [8]. High levels of the stress adaptor protein p66^shc^ and elevated reactive oxygen species (ROS) further drive this phenotype, which can be induced by high oxygen tensions in the culture environment in permanently arrested early embryos [61].

The repression of AURKA has been identified as a cause of arrest. AURKA is essential for microtubule cytoskeletal organization; its inhibition leads to distorted spindles (multipolar or diffuse) and increased genomic instability [7]. Furthermore, AURKA repression impedes glycolysis, causing embryos to erroneously maintain an oxidative phosphorylation-biased metabolism that cannot meet the energy demands of compaction and blastulation [7]. Notably, treatment with SIRT agonists like resveratrol or nicotinamide riboside (NR) can push embryos toward a more glycolytic state and partially rescue the arrested phenotype by modulating metabolic activity and overcome senescence [8].

### 3.6. Chromosome Abnormalities and Aneuploidy

Transcriptomic and epigenetic factors are critical, but chromosomal aneuploidy was also a key cause of developmental arrest in the early embryos, particularly in women with advanced age [62]. Euploid embryos have a significantly higher chance to reach the blastocyst stage compared to aneuploid ones [63]. Nearly 70% of arrested blastomere biopsies exhibit chromosomal errors by using fluorescence in situ hybridization (FISH), including polyploidy, haploidy, and chaotic complements. Although aneuploid embryos can develop into the blastocyst stage at relatively high rates, they remain a major cause of implantation failure [64,65].

The prevalence of complex/multiple chromosomal abnormalities is significantly higher in arrested embryos than in developing blastocysts. These errors arose from spindle disorganization, deterioration of sister chromatid cohesion, and failure of the spindle assembly checkpoint during oocyte maturation and early mitosis [66]. Although some aneuploid embryos can reach the blastocyst stage, the high rate of chaotic division in arrested embryos suggests that severe genomic imbalances are incompatible with the metabolic and transcriptomic transitions required for preimplantation survival [63].

### 3.7. Multi-Omics Crosstalk in Human PREMBA

The transition from oocyte to embryo is a highly orchestrated symphony of molecular events, where the failure of any omics can lead to a catastrophic “molecular traffic jam” resulting in developmental arrest (Table 2 and Figure 1). The center of this process is the MZT, a milestone characterized by the degradation of maternal transcripts and the activation of ZGA. Recent single-cell multi-omics evidence identifies that the “transcriptionally arrested status” (TAS) represents a significant portion of morphologically normal 8-cell embryos, quantified by an abnormally high maternal allelic ratio (MAR). This transcriptomic stagnation is primarily driven by the deficiency of primate-specific PRD-like homeobox transcription factors: specifically, DPRX and ARGFX [6] and the master regulator YY1 [7].

The failure to activate these factors creates an immediate epigenetic “lock”, where the downregulation of deacetylases such as HDAC1 lead to the aberrant retention of H3K27ac modifications. This epigenetic rigidity prevents the timely silencing of 4-cell-specific genes like *LEUTX* and *CCNA1*, effectively trapping the embryo into an outdated developmental program and preventing the necessary shift toward lineage specification [6]. Simultaneously, the failure of KDM5B-mediated H3K4me3 reprogramming from broad to canonical patterns further hinders the initiation of the major ZGA program [7].

The secondary layer of multi-omics crosstalk involves the synergy between autophagic pathways and the chromatin landscape, which is essential for maintaining genomic stability. Successful MZT requires the rapid clearance of “maternal inheritance” through two distinct pathways: (1) the canonical deadenylase-mediated degradation (e.g., BTG4 and CNOT6L) and (2) the newly identified LC3B-dependent autophagy pathway.

LC3B acts as an RNA-binding protein that preferentially targets maternal Z-decay mRNAs, such as the methyltransferase Suv39h2, for lysosomal degradation [45]. When this autophagic crosstalk is inhibited, the accumulation of maternal Suv39h2 results in increased H3K9me3 levels at ZGA hubs, particularly mouse ERVL (MERVL) retrotransposons, which act as a repressive barrier to the “awakening” of the zygotic genome [53]. This failure in maternal clearance is often coupled with a disorganized chromatin state where chromatin accessibility (NDRs) outruns the transcriptome, occurring independently of cell division. Such misplaced openness leads to the massive de-repression of TEs, including the Alu, SVA, and LINE-1 families, which triggers genomic instability and DNA damage—evidenced by elevated γ-H2AX foci [7].

Ultimately, these transcriptomic and epigenetic failures converge into a terminal physiological phenotype characterized by metabolic collapse and cytoskeletal distortion, pushing the embryos into a senescent-like state [8]. Multi-omics profiling has identified the repression of AURKA as a critical node in this process, as it simultaneously disrupts microtubule organization—leading to multipolar spindles—and impedes glycolysis by promoting the hypermethylation state and silencing of key metabolic genes such as *SLC2A3* and lactate dehydrogenase A (*LDHA*) [7].

Consequently, arrested embryos remain trapped in an oxidative phosphorylation-biased metabolism that fails to meet the high energy demands required for compaction and blastulation. This state is further driven by the activation of the p53/p21 (*CDKN1A*) pathway and the stress adaptor protein p66^shc^, which accumulates in response to high ROS levels [61]. Intriguingly, the crosstalk between metabolism and the epigenome offers therapeutic potential; treatments with SIRT agonists like resveratrol or the HDAC1 activator exifone can partially rescue the arrested phenotype by restoring metabolic balance and erasing aberrant histone marks [8]. By bridging these omics dimensions, it becomes clear that human early embryonic arrest is not a singular defect but the outcome of a systemic failure in the molecular feedback loops required to synchronize the genome with the metabolic and structural demands of life (Figure 1).

**Table 2:** Genes/TFs/TEs associated with embryonic arrest for unexplained reasons.

Type	Abnormal Program	Arrested Stage	Related Genes/TFs/TEs
Transcriptomic dysregulation of MZT	M-decay	Zygote	*BTG4*, *CNOT6L*, *CNOT7*
	Major ZGA	8-cell and zygote to 4-cell	DPRX, ARGFX and YY1
	Z-decay	8-cell	*TUT4/7*
Abnormal DNA methylation reprogramming	Demethylation	-	TET family, *DNMT1* and *UHRF1*
Chromatin accessibility and genomic instability	Establishment of TADs	Zygote to 8-cell	CTCF
Defective histone modification dynamics	H3K4me3	Zygote to 8-cell	*KDM5B*
	H3K9me3	8-cell	MLT2A1
	H3K27ac	8-cell	*HDAC1*, *HDAC2*, and *SIRT1*
Metabolic shifts, cytoskeletal distortion and senescence	Microtubule cytoskeletal organization	Zygote and 2-cell	*AURKA*
	Glycolysis	Fertilization to 8-cell	*SIRT1*, *AURKA* and *SLC2A3*

MZT, maternal-to-zygotic transition; M-decay, maternal RNA decay; ZGA, zygotic genome activation; Z-decay, zygotic mRNA decay; TADs, topologically associating domains; H3K4me3, histone H3 lysine 4 trimethylation; H3K9me3, histone H3 lysine 9 trimethylation; H3K27ac, histone H3 lysine 27 acetylation; *BTG4*, BTG anti-proliferation factor 4; *CNOT6L*, CCR4-NOT transcription complex subunit 6 like; *CNOT7*, CCR4-NOT transcription complex subunit 7; DPRX, divergent-paired related homeobox; ARGFX, arginine-fifty homeobox; YY1, Yin Yang 1; *TUT4/7*, terminal uridylyl transferase 4/7; TET, Tet methylcytosine dioxygenase; *DNMT1*, DNA methyltransferase 1; *UHRF1*, ubiquitin like with PHD and ring finger domains 1; CTCF, CCCTC-binding factor; *KDM5B*, lysine demethylase 5B; MLT2A1, a human totipotency-specific long terminal repeat (LTR) of ERVL subfamily; *HDAC1/2*, histone deacetylase 1/2; *SIRT1*, sirtuin 1; *AURKA*, aurora kinase A; and *SLC2A3*, solute carrier family 2 member 3.

## 4. Conclusions and Future Outlook

The studies on human preimplantation embryo arrest have advanced from simple morphological descriptions to a sophisticated molecular dissection of the genetic landscape and multi-omics dysregulation. Currently, PREMBA is a primary cause of recurrent IVF or intracytoplasmic sperm injection (ICSI) failure, with approximately 10% of IVF embryos undergoing arrest at the cleavage stages [1] and around 60% of embryos failed to reach the blastocyst stage [67]. This review has categorized the etiology of embryonic arrest into two major classes: known genetic mutations and those with unexplained reasons. We provide a comprehensive summary of currently known genetic mutations associated with PREMBA, along with the latest advances in multi-omics studies on non-genomic embryonic arrest (Figure 1).

Mutations in genes like *BTG4* and *CHEK1* result in zygotic cleavage failures by disrupting MZT; specifically, *BTG4* mutations prevent the programmed degradation of maternal mRNAs [10], while *CHEK1* gain-of-function variants cause an abnormal accumulation of inhibitory phosphorylated CDK1, leading to pronuclear fusion failure [5]. Defects in the SCMC, involving genes such as *PADI6* [20], *TLE6* [21], and the *NLRP* family [24], impair ZGA, symmetry of cleavage, and genomic stability, typically causing embryos to arrest at the 2- to 8-cell or morula stages. The failure of nuclear protein transport, primarily due to *KPNA7* [4] deficiency, prevents the import of critical ZGA-triggering factors like RSL1D1 into the nucleus, effectively stalling the developmental program. Cell cycle and cytoskeletal regulators, including *FBXO43* [38] and tubulin genes like *TUBA4A* and *TUBB8*, are essential for spindle assembly and the stabilization of Cyclin B1; mutations in these genes exert dominant negative effects that destabilize the microtubule network and cause severe embryo fragmentation [35]. Finally, errors in meiotic recombination and DNA repair genes, such as *REC114* and *MEI1*, leading to severe aneuploidy and abnormal pronuclear formation [31].

MZT failure was a primary non-genetic etiology. This status is often driven by the deficiency of primate-specific PRD-like homeobox genes (*DPRX* and *ARGFX*) [6] and the master transcription factor YY1 [7], leading to catastrophic failures in major zygotic genome activation. These transcriptomic blocks are intricately linked to epigenetic reprogramming defects, including the DNA hypermethylation state that silences metabolic genes like *SLC2A3*, and the aberrant retention of H3K4me3, H3K9me3 or H3K27ac patterns [6]. Such molecular disorders frequently force embryos into a “senescent-like” state characterized by the activation of the p53/p21 pathway, ribosome downregulation, and a failed metabolic shift from oxidative phosphorylation to glycolysis. Furthermore, recent evidence highlights the repression of AURKA as a critical node, causing microtubule cytoskeletal distortion, spindle assembly defects, and increased genomic instability [7]. Emerging studies suggest that these developmental blocks can be partially bypassed using SIRT agonists or HDAC1 activators, which help to restore metabolic balance and erase aberrant histone marks, offering new potential strategies for improving clinical ART success rates [8].

Advances in WES will enable the identification of the additional gene mutations responsible for embryonic arrest. Furthermore, the application of multi-omics sequencing technologies promises to elucidate the molecular mechanisms underlying currently unexplained cases of developmental arrest through multidimensional omics analyses. That will not only provide a deeper understanding of embryonic arrest etiology but also facilitate clinical intervention design, ultimately improving embryo developmental competence and oocyte utilization efficiency.

Future breakthroughs in understanding and managing PREMBA require a multifaceted approach. The pivotal advancement is the integration of artificial intelligence (AI) and time-lapse imaging to improve the early identification of embryos at high risk of arrest. By utilizing computational machine learning to detect subtle morphokinetic markers non-invasively before the major ZGA, clinicians can better predict developmental competence and optimize embryo selection.

The expansion of multi-omics research—integrating genomics, transcriptomics, epigenomics, and metabolomics—will be essential to decipher the molecular mechanisms behind the arrest cases for unexplained reasons. Recently, RNA modifications, such as RNA N^6^-methyladenosine (m^6^A) and pseudouridylation (Ψ), have been shown to play pivotal roles in diverse biological processes [68]. However, their involvement in human embryonic developmental arrest remains poorly characterized.

Moreover, current mechanistic investigations of human embryonic arrest have predominantly focused on the MZT process, including M-decay, ZGA, and Z-decay. However, protein-coding genes constitute only a minor fraction of the human genome. Numerous non-coding RNAs (ncRNAs)—including microRNAs (miRNAs), long non-coding RNAs (lncRNAs), circular RNAs (circRNAs), and PIWI-interacting RNAs (piRNAs)—have been demonstrated to play crucial roles in embryonic development [69,70,71]. Their potential involvement in human embryonic arrest remains significantly underexplored.

Additionally, resolving the molecular etiology of PREMBA will require systematic interrogation of multi-omics crosstalk in the future. While current studies have independently mapped genomic aberrations, transcriptomic dysregulation, and epigenetic landscapes in arrested embryos, integrative analyses remain scarce. Emerging single-cell multi-omics technologies, particularly simultaneous genome–transcriptome–epigenome profiling, promise to unveil regulatory networks underlying developmental competence. This enables a multidimensional understanding of embryonic developmental arrest causes, thereby facilitating more targeted strategies to improve IVF outcomes.

## Data Availability

No new data were created or analyzed in this study. Data sharing is not applicable to this article.

## References

[1] Liu L., Cai J., Li P., Chen Y., Sha A., Ren J. (2016). Clinical Outcome of IVF/ICSI Cycles with an Arrested Embryo on Day 3. Int. J. Clin. Exp. Med..

[2] Wei Y., Wang J., Qu R., Zhang W., Tan Y., Sha Y., Li L., Yin T. (2024). Genetic Mechanisms of Fertilization Failure and Early Embryonic Arrest: A Comprehensive Review. Hum. Reprod. Update.

[3] Wang B. (2025). Genetic Landscape of Human Oocyte/Embryo Defects. Cell Genom..

[4] Wang W., Miyamoto Y., Chen B., Shi J., Diao F., Zheng W., Li Q., Yu L., Li L., Xu Y. (2023). Karyopherin α Deficiency Contributes to Human Preimplantation Embryo Arrest. J. Clin. Investig..

[5] Zhang H., Chen T., Wu K., Hou Z., Zhao S., Zhang C., Gao Y., Gao M., Chen Z.-J., Zhao H. (2021). Dominant Mutations in CHK1 Cause Pronuclear Fusion Failure and Zygote Arrest That Can Be Rescued by CHK1 Inhibitor. Cell Res..

[6] Guo Q., Xu F., Song S., Kong S., Zhai F., Xiu Y., Liu D., Li M., Lian Y., Ding L. (2025). Allelic Transcriptomic Profiling Identifies the Role of PRD-like Homeobox Genes in Human Embryonic-Cleavage-Stage Arrest. Dev. Cell.

[7] Wang T., Peng J., Fan J., Tang N., Hua R., Zhou X., Wang Z., Wang L., Bai Y., Quan X. (2024). Single-Cell Multi-Omics Profiling of Human Preimplantation Embryos Identifies Cytoskeletal Defects during Embryonic Arrest. Nat. Cell Biol..

[8] Yang Y., Shi L., Fu X., Ma G., Yang Z., Li Y., Zhou Y., Yuan L., Xia Y., Zhong X. (2022). Metabolic and Epigenetic Dysfunctions Underlie the Arrest of in Vitro Fertilized Human Embryos in a Senescent-like State. PLoS Biol..

[9] Yu C., Ji S.-Y., Sha Q.-Q., Dang Y., Zhou J.-J., Zhang Y.-L., Liu Y., Wang Z.-W., Hu B., Sun Q.-Y. (2016). BTG4 Is a Meiotic Cell Cycle–Coupled Maternal-Zygotic-Transition Licensing Factor in Oocytes. Nat. Struct. Mol. Biol..

[10] Zheng W., Zhou Z., Sha Q., Niu X., Sun X., Shi J., Zhao L., Zhang S., Dai J., Cai S. (2020). Homozygous Mutations in BTG4 Cause Zygotic Cleavage Failure and Female Infertility. Am. J. Hum. Genet..

[11] Zheng W., Sha Q.-Q., Hu H., Meng F., Zhou Q., Chen X., Zhang S., Gu Y., Yan X., Zhao L. (2022). Biallelic Variants in ZFP36L2 Cause Female Infertility Characterised by Recurrent Preimplantation Embryo Arrest. J. Med. Genet..

[12] Rong Y., Ji S.-Y., Zhu Y.-Z., Wu Y.-W., Shen L., Fan H.-Y. (2019). ZAR1 and ZAR2 Are Required for Oocyte Meiotic Maturation by Regulating the Maternal Transcriptome and mRNA Translational Activation. Nucleic Acids Res..

[13] Miao L., Yuan Y., Cheng F., Fang J., Zhou F., Ma W., Jiang Y., Huang X., Wang Y., Shan L. (2017). Translation Repression by Maternal RNA Binding Protein Zar1 Is Essential for Early Oogenesis in Zebrafish. Development.

[14] Lin Y., Protter D.S.W., Rosen M.K., Parker R. (2015). Formation and Maturation of Phase Separated Liquid Droplets by RNA Binding Proteins. Mol. Cell.

[15] Wu X., Viveiros M.M., Eppig J.J., Bai Y., Fitzpatrick S.L., Matzuk M.M. (2003). Zygote Arrest 1 (Zar1) Is a Novel Maternal-Effect Gene Critical for the Oocyte-to-Embryo Transition. Nat. Genet..

[16] Tian Y., Yang J., Peng Y., Chen T., Huang T., Zhang C., Zhao H. (2020). Variation Screening of Zygote Arrest 1(ZAR1) in Women with Recurrent Zygote Arrest during IVF/ICSI Programs. Reprod. Sci..

[17] Liao X.-Q., Zhao S., Zhang C.-L., Zhu Y., Wen J., Wang Y., Liu S.-Y., Su W., Zhang J.-T., Lu Q.-W. (2025). ZAR1 Pathogenic Variants Disrupt Maternal mRNA Storage and Cause Oocyte Maturation Defects in Humans. Cell. Mol. Life Sci..

[18] Jentoft I.M.A., Bäuerlein F.J.B., Welp L.M., Cooper B.H., Petrovic A., So C., Penir S.M., Politi A.Z., Horokhovskyi Y., Takala I. (2023). Mammalian Oocytes Store Proteins for the Early Embryo on Cytoplasmic Lattices. Cell.

[19] Bebbere D., Albertini D.F., Coticchio G., Borini A., Ledda S. (2021). The Subcortical Maternal Complex: Emerging Roles and Novel Perspectives. Mol. Hum. Reprod..

[20] Xu Y., Shi Y., Fu J., Yu M., Feng R., Sang Q., Liang B., Chen B., Qu R., Li B. (2016). Mutations in PADI6 Cause Female Infertility Characterized by Early Embryonic Arrest. Am. J. Hum. Genet..

[21] Alazami A.M., Awad S.M., Coskun S., Al-Hassan S., Hijazi H., Abdulwahab F.M., Poizat C., Alkuraya F.S. (2015). TLE6 Mutation Causes the Earliest Known Human Embryonic Lethality. Genome Biol..

[22] Tong X., Jin J., Hu Z., Zhang Y., Fan H., Zhang Y., Zhang S. (2022). Mutations in OOEP and NLRP5 Identified in Infertile Patients with Early Embryonic Arrest. Hum. Mutat..

[23] Akoury E., Zhang L., Ao A., Slim R. (2015). NLRP7 and KHDC3L, the Two Maternal-Effect Proteins Responsible for Recurrent Hydatidiform Moles, Co-Localize to the Oocyte Cytoskeleton. Hum. Reprod..

[24] Mu J., Wang W., Chen B., Wu L., Li B., Mao X., Zhang Z., Fu J., Kuang Y., Sun X. (2019). Mutations in NLRP2 and NLRP5 Cause Female Infertility Characterised by Early Embryonic Arrest. J. Med. Genet..

[25] Sills E.S., Obregon-Tito A.J., Gao H., McWilliams T.K., Gordon A.T., Adams C.A., Slim R. (2017). Pathogenic Variant in NLRP7 (19q13.42) Associated with Recurrent Gestational Trophoblastic Disease: Data from Early Embryo Development Observed during in Vitro Fertilization. Clin. Exp. Reprod. Med..

[26] Vitale A., Yurttas P., Fitzhenry R., Cohen-Gould L., Wu W., Gossen J., Coonrod S. (2008). Role for PADI6 and the CPLs in Ribosomal Storage in Oocytes and Translation in the Early Embryo. Development.

[27] Libby B.J., Reinholdt L.G., Schimenti J.C. (2003). Positional Cloning and Characterization of *Mei1*, a Vertebrate-Specific Gene Required for Normal Meiotic Chromosome Synapsis in Mice. Proc. Natl. Acad. Sci. USA.

[28] Kumar R., Ghyselinck N., Ishiguro K., Watanabe Y., Kouznetsova A., Höög C., Strong E., Schimenti J., Daniel K., Toth A. (2015). MEI4—A Central Player in the Regulation of Meiotic DNA Double-strand Break Formation in the Mouse. J. Cell Sci..

[29] Laroussi H., Juarez-Martinez A.B., Le Roy A., Boeri Erba E., Gabel F., de Massy B., Kadlec J. (2023). Characterization of the REC114-MEI4-IHO1 Complex Regulating Meiotic DNA Double-strand Break Formation. EMBO J..

[30] Kumar R., Oliver C., Brun C., Juarez-Martinez A.B., Tarabay Y., Kadlec J., de Massy B. (2018). Mouse REC114 Is Essential for Meiotic DNA Double-Strand Break Formation and Forms a Complex with MEI4. Life Sci. Alliance.

[31] Wang W., Dong J., Chen B., Du J., Kuang Y., Sun X., Fu J., Li B., Mu J., Zhang Z. (2020). Homozygous Mutations in REC114 Cause Female Infertility Characterised by Multiple Pronuclei Formation and Early Embryonic Arrest. J. Med. Genet..

[32] Wang K., Sturt-Gillespie B., Hittle J.C., Macdonald D., Chan G.K., Yen T.J., Liu S.-T. (2014). Thyroid Hormone Receptor Interacting Protein 13 (TRIP13) AAA-ATPase Is a Novel Mitotic Checkpoint-Silencing Protein. J. Biol. Chem..

[33] Hu H., Zhang S., Guo J., Meng F., Chen X., Gong F., Lu G., Zheng W., Lin G. (2022). Identification of Novel Variants of Thyroid Hormone Receptor Interaction Protein 13 That Cause Female Infertility Characterized by Zygotic Cleavage Failure. Front. Physiol..

[34] Zhu J.-L., Liang X. (2025). TUBA4A: The Tale of an Unconventional Tubulin. Cytoskeleton.

[35] Li Q., Zhao L., Zeng Y., Kuang Y., Guan Y., Chen B., Xu S., Tang B., Wu L., Mao X. (2023). Large-Scale Analysis of de Novo Mutations Identifies Risk Genes for Female Infertility Characterized by Oocyte and Early Embryo Defects. Genome Biol..

[36] Cao T., Guo J., Xu Y., Lin X., Deng W., Cheng L., Zhao H., Jiang S., Gao M., Huang J. (2021). Two Mutations in TUBB8 Cause Developmental Arrest in Human Oocytes and Early Embryos. Reprod. Biomed. Online.

[37] Feng R., Yan Z., Li B., Yu M., Sang Q., Tian G., Xu Y., Chen B., Qu R., Sun Z. (2016). Mutations in TUBB8 Cause a Multiplicity of Phenotypes in Human Oocytes and Early Embryos. J. Med. Genet..

[38] Wang W., Wang W., Xu Y., Shi J., Fu J., Chen B., Mu J., Zhang Z., Zhao L., Lin J. (2021). FBXO43 Variants in Patients with Female Infertility Characterized by Early Embryonic Arrest. Hum. Reprod..

[39] Zhang Y.-L., Zheng W., Ren P., Jin J., Hu Z., Liu Q., Fan H.-Y., Gong F., Lu G.-X., Lin G. (2022). Biallelic Variants in MOS Cause Large Polar Body in Oocyte and Human Female Infertility. Hum. Reprod..

[40] Zhao L., Xue S., Yao Z., Shi J., Chen B., Wu L., Sun L., Xu Y., Yan Z., Li B. (2020). Biallelic Mutations in CDC20 Cause Female Infertility Characterized by Abnormalities in Oocyte Maturation and Early Embryonic Development. Protein Cell.

[41] Zhao L., Guan Y., Meng Q., Wang W., Wu L., Chen B., Hu J., Zhu J., Zhang Z., Mu J. (2021). Identification of Novel Mutations in CDC20: Expanding the Mutational Spectrum for Female Infertility. Front. Cell Dev. Biol..

[42] Huang L., Wang F., Kong S., Wang Y., Song G., Lu F., Ji J., Luo L., Tong X. (2021). Novel Mutations in CDC20 Are Associated with Female Infertility Due to Oocyte Maturation Abnormality and Early Embryonic Arrest. Reprod. Sci..

[43] Sha Q.-Q., Zhang J., Fan H.-Y. (2019). A Story of Birth and Death: MRNA Translation and Clearance at the Onset of Maternal-to-Zygotic Transition in Mammals. Biol. Reprod..

[44] Sha Q.-Q., Zheng W., Wu Y.-W., Li S., Guo L., Zhang S., Lin G., Ou X.-H., Fan H.-Y. (2020). Dynamics and Clinical Relevance of Maternal mRNA Clearance during the Oocyte-to-Embryo Transition in Humans. Nat. Commun..

[45] Liu D., Guo S., Hu J., Zhu L., Wang J., Yang S., Zhang Y., Huang G., Gao S., Zhu Q. (2026). Autophagy Regulates the Maternal-to-Zygotic Transition through MAP1LC3B-Mediated Maternal mRNA Decay. Autophagy.

[46] Hwang H.J., Ha H., Lee B.S., Kim B.H., Song H.K., Kim Y.K. (2022). LC3B Is an RNA-Binding Protein to Trigger Rapid mRNA Degradation during Autophagy. Nat. Commun..

[47] Jukam D., Shariati S.A.M., Skotheim J.M. (2017). Zygotic Genome Activation in Vertebrates. Dev. Cell.

[48] Zhu P., Guo H., Ren Y., Hou Y., Dong J., Li R., Lian Y., Fan X., Hu B., Gao Y. (2018). Single-Cell DNA Methylome Sequencing of Human Preimplantation Embryos. Nat. Genet..

[49] Arand J., Reijo Pera R.A., Wossidlo M. (2021). Reprogramming of DNA Methylation Is Linked to Successful Human Preimplantation Development. Histochem. Cell Biol..

[50] Hernandez Mora J.R., Buhigas C., Clark S., Del Gallego Bonilla R., Daskeviciute D., Monteagudo-Sánchez A., Poo-Llanillo M.E., Medrano J.V., Simón C., Meseguer M. (2023). Single-Cell Multi-Omic Analysis Profiles Defective Genome Activation and Epigenetic Reprogramming Associated with Human Pre-Implantation Embryo Arrest. Cell Rep..

[51] Gerdes P., Richardson S.R., Mager D.L., Faulkner G.J. (2016). Transposable Elements in the Mammalian Embryo: Pioneers Surviving through Stealth and Service. Genome Biol..

[52] Wu J., Xu J., Liu B., Yao G., Wang P., Lin Z., Huang B., Wang X., Li T., Shi S. (2018). Chromatin Analysis in Human Early Development Reveals Epigenetic Transition during ZGA. Nature.

[53] Xiang Y., Qian Y., Li Z., Wang J., Tian R., Meng W., Bu J., Huang F., Ai Z., Wu D. (2026). Endogenous Retroviruses Synthesize Heterologous Chimeric RNAs to Reinforce Human Early Embryo Development. Science.

[54] Chen X., Ke Y., Wu K., Zhao H., Sun Y., Gao L., Liu Z., Zhang J., Tao W., Hou Z. (2019). Key Role for CTCF in Establishing Chromatin Structure in Human Embryos. Nature.

[55] Xia W., Xu J., Yu G., Yao G., Xu K., Ma X., Zhang N., Liu B., Li T., Lin Z. (2019). Resetting Histone Modifications during Human Parental-to-Zygotic Transition. Science.

[56] Shen H., Xu W., Lan F. (2017). Histone Lysine Demethylases in Mammalian Embryonic Development. Exp. Mol. Med..

[57] Wilkinson A.L., Zorzan I., Rugg-Gunn P.J. (2023). Epigenetic Regulation of Early Human Embryo Development. Cell Stem Cell.

[58] Xu R. (2022). Stage-Specific H3K9me3 Occupancy Ensures Retrotransposon Silencing in Human Pre-Implantation Embryos. Cell Stem Cell.

[59] Sankar A., Lerdrup M., Manaf A., Johansen J.V., Gonzalez J.M., Borup R., Blanshard R., Klungland A., Hansen K., Andersen C.Y. (2020). KDM4A Regulates the Maternal-to-Zygotic Transition by Protecting Broad H3K4me3 Domains from H3K9me3 Invasion in Oocytes. Nat. Cell Biol..

[60] Wu K., Fan D., Zhao H., Liu Z., Hou Z., Tao W., Yu G., Yuan S., Zhu X., Kang M. (2023). Dynamics of Histone Acetylation during Human Early Embryogenesis. Cell Discov..

[61] Favetta L.A., John E.J.S., King W.A., Betts D.H. (2007). High Levels of P66shc and Intracellular ROS in Permanently Arrested Early Embryos. Free. Radic. Biol. Med..

[62] Rienzi L., Cimadomo D., Delgado A., Minasi M.G., Fabozzi G., del Gallego R., Stoppa M., Bellver J., Giancani A., Esbert M. (2019). Time of Morulation and Trophectoderm Quality Are Predictors of a Live Birth after Euploid Blastocyst Transfer: A Multicenter Study. Fertil. Steril..

[63] Maurer M., Ebner T., Puchner M., Mayer R.B., Shebl O., Oppelt P., Duba H.-C. (2015). Chromosomal Aneuploidies and Early Embryonic Developmental Arrest. Int. J. Fertil. Steril..

[64] Shahbazi M.N., Wang T., Tao X., Weatherbee B.A.T., Sun L., Zhan Y., Keller L., Smith G.D., Pellicer A., Scott R.T. (2020). Developmental Potential of Aneuploid Human Embryos Cultured beyond Implantation. Nat. Commun..

[65] Orvieto R., Jonish-Grossman A., Maydan S.A., Noach-Hirsh M., Dratviman-Storobinsky O., Aizer A. (2022). Cleavage-Stage Human Embryo Arrest, Is It Embryo Genetic Composition or Others?. Reprod. Biol. Endocrinol..

[66] Qi S.-T., Liang L.-F., Xian Y.-X., Liu J.-Q., Wang W. (2014). Arrested Human Embryos Are More Likely to Have Abnormal Chromosomes than Developing Embryos from Women of Advanced Maternal Age. J. Ovarian Res..

[67] Wong C.C., Loewke K.E., Bossert N.L., Behr B., De Jonge C.J., Baer T.M., Pera R.A.R. (2010). Non-Invasive Imaging of Human Embryos before Embryonic Genome Activation Predicts Development to the Blastocyst Stage. Nat. Biotechnol..

[68] Li Y., Wang Y., Cengiz A., Jin K.-X., Castroviejo B.C., Lin X., Indahl M., Zuo R., Skuland T., Fosslie M. (2025). The RNA m6A Landscape during Human Oocyte-to-Embryo Transition. EMBO J..

[69] Xu W., Liu J., Qi H., Si R., Zhao Z., Tao Z., Bai Y., Hu S., Sun X., Cong Y. (2024). A Lineage-Resolved Cartography of microRNA Promoter Activity in C. Elegans Empowers Multidimensional Developmental Analysis. Nat. Commun..

[70] Russell S.J., Zhao C., Biondic S., Menezes K., Hagemann-Jensen M., Librach C.L., Petropoulos S. (2024). An Atlas of Small Non-Coding RNAs in Human Preimplantation Development. Nat. Commun..

[71] Verma A., Ghosh B., Das T., Deb A., Mukherjee A., Sengupta P., Sarkar A., Jana K., Ghosh Z. (2025). Parental Noncoding RNA Expression Dynamics across Sperm, Oocyte, and Zygote. NAR Genom. Bioinform..

